# *Borrelia burgdorferi* and autoimmune mechanisms: implications for mimicry, misdiagnosis, and mismanagement in Lyme disease and autoimmune disorders

**DOI:** 10.1007/s00296-024-05580-x

**Published:** 2024-04-05

**Authors:** Bohdana Doskaliuk, Olena Zimba

**Affiliations:** 1 Department of Patophysiology, Ivano-Frankivsk National Medical University, Halytska str. 2, Ivano-Frankivsk, 76000 Ukraine; 2 Department of Clinical Rheumatology and Immunology, University Hospital in Krakow, Kraków, Poland; 3 National Institute of Geriatrics, Rheumatology and Rehabilitation, Warsaw, Poland; 4 Department of Internal Medicine N2, Danylo Halytsky Lviv National Medical University, Lviv, Ukraine

**Keywords:** Borrelia, Scleroderma, Systemic lupus erythematosus, Dermatomyositis, Molecular mimicry

## Abstract

The genus Borrelia encompasses a diverse group of spirochetes transmitted primarily by ticks, with *Borrelia burgdorferi* causing Lyme disease, which is prevalent in North America and Europe. Borrelia’s structural adaptations and ability to persist in diverse host tissues underscore its pathogenic potential. Beyond traditional infectious responses, Borrelia engages in complex interactions with the host immune system, contributing to autoimmune mechanisms such as molecular mimicry and persistent infections. This intricate interplay manifests in symptoms resembling various autoimmune diseases, including systemic lupus erythematosus, dermatomyositis, local scleroderma, and systemic sclerosis. However, these associations lack a precise explanation, emphasizing the need for further investigation. The cases of misdiagnosis between Lyme borreliosis and autoimmune diseases highlight the critical importance of accurate diagnostics and adherence to guidelines. Understanding Borrelia’s impact on immune responses is pivotal for advancing diagnostics and targeted therapeutic interventions in Lyme borreliosis and its potential autoimmune implications.

## Introduction

Borrelia, a genus of spirochetes, comprises a diverse group of bacteria known for their unique spiral shape and complex life cycle [1]. These microorganisms are primarily transmitted through arthropod vectors, particularly ticks, and have been implicated in various human diseases. Spirochetes are characterized by their spiral-shaped morphology, facilitated by axial filaments that run longitudinally along the bacteria’s body [2]. Borrelia spirochetes are motile, exhibiting a corkscrew motion, enabling them to navigate diverse host tissues and evade the host’s immune system. This unique structural adaptation contributes to the pathogenicity and persistence of Borrelia within the host. Borrelia species share standard features that distinguish them from other bacteria. They possess a segmented genome, contributing to their genomic plasticity. In addition, Borrelia can alter their surface proteins, facilitating immune evasion [3]. The ability to persist in diverse host tissues, including joints, skin, and the central nervous system, underlines their adaptability and pathogenic potential [4].

Borrelia spirochetes are worldwide, showing different prevalence levels across various regions. The most recognized species, *Borrelia burgdorferi*, known for causing Lyme borreliosis (LB), has a notably high prevalence in North America, Europe, and some areas of Asia [5].

In the most recent national analysis of insurance claims data from 2010 to 2018, researchers estimated that approximately 476,000 patients are diagnosed and treated for LB each year in the United States [6]. In Europe, LB is widespread but inconsistent, with annual rates fluctuating between 1 and 100 cases per 100,000 individuals. In addition, there are countries where LB is rapidly emerging as a significant health concern, such as Canada and Ukraine [6, 7].

In addition, different Borrelia species like *Borrelia afzelii* and *Borrelia garinii* add to the diversity of Lyme borreliosis (LB) in specific geographical locations.

*Borrelia burgdorferi*: This species is the primary cause of LB and is prevalent in North America and Europe. It exhibits significant genetic diversity, contributing to variations in disease manifestations.

*Borrelia afzelii*: Predominantly found in European ticks, this species is associated with cutaneous manifestations of LB and is often implicated in acrodermatitis chronica atrophicans [8].

*Borrelia garinii*: Prevalent in Europe and Asia, *B. garinii* is frequently associated with neuroborreliosis, leading to diverse neurological symptoms [5].

*Borrelia mayonii*: Recently identified in North America, this species is responsible for a subset of Lyme disease cases, displaying unique genetic characteristics [9].

*Borrelia recurrentis*: Causative agent of relapsing fever, transmitted by body lice, *B. recurrentis* poses a threat in regions with poor living conditions [10].

*Borrelia hermsii, Borrelia turicatae*, and others: These species contribute to tick-borne relapsing fever and are prevalent in different parts of North America [11].

Therefore, the objective of this manuscript is to investigate and clarify the intricate interactions between *Borrelia burgdorferi* and host immune responses. It further aims to assess how these interactions may lead to autoimmune reactions and the possibility that Borrelia can replicate symptoms of autoimmune rheumatic diseases.

## Search strategy

A literature review was carried out across databases, such as Scopus and MEDLINE/PubMed, using a range of MeSH search terms. Keywords used in the search included “borrelia”, “Lyme disease”, “borreliosis”, “rheumatic disease”, “systemic sclerosis”, “scleroderma”, “morphea”, “systemic lupus erythematosus”, “dermatomyositis”, and “autoimmunity”. Each article identified was carefully assessed for its relevance. In addition, the references of these articles were examined to find any additional related sources. The review focused on peer-reviewed articles published in English without setting the time limit for search. To ensure the review’s reliability and relevance, specific criteria for exclusion were applied following the initial search phase. These criteria excluded articles without an abstract, document types like reviews, book chapters, conference proceedings, and errata, retracted articles, and studies not highlighting the possible connection between borreliosis and systemic autoimmune connective tissue diseases.

## Borrelia and autoimmunity

The pathogenesis of Borrelia infection unfolds beyond the conventional scope of infectious diseases, delving into a complex interplay with the host immune system, ultimately contributing to autoimmune mechanisms. Upon transmission through tick bites, Borrelia adopts a stealthy invasion strategy, disseminating throughout the host while adeptly evading immune detection via alterations in surface proteins [12]. The remarkable antigenic variation of outer surface proteins (Osps) empowers Borrelia to elude immune recognition, thereby establishing a persistent infection [12].

Triggering responses from the host’s innate and adaptive immune systems, Borrelia infection prompts the recruitment of macrophages and neutrophils to the site of infection to eliminate the spirochetes [13]. However, Borrelia employs various evasion tactics, including downregulating complement activation and resistance to phagocytosis, thereby prolonging its survival within the host.

Recent findings have shed light on a link between infection by Borrelia and the onset of autoimmune conditions. Molecular mimicry is at the forefront of these autoimmune mechanisms, wherein spirochetal antigens exhibit structural similarities to host antigens, leading to cross-reactive immune responses [14]. This phenomenon may catalyze the onset or exacerbation of autoimmune conditions. The ensuing production of autoantibodies, particularly those targeting host tissues such as joints and neural structures, further contributes to the complex landscape of autoimmune responses. Notably, in diseases like rheumatoid arthritis, anti-Borrelia antibodies have been implicated in joint pathology [15, 16].

Persistent infections established by Borrelia contribute to chronic inflammation, a hallmark of autoimmune diseases. By evading clearance mechanisms and residing in immune-privileged sites, spirochetes perpetuate the inflammatory response, creating an environment conducive to autoimmunity.

## Borrelia—a great mimicker

Lyme borreliosis can present a wide range of clinical symptoms encompassing dermatological, cardiac, joint, neurological, cardiovascular, and ocular issues [17]. These symptoms often closely resemble those seen in various autoimmune pathologies, including rheumatoid arthritis (RA), systemic sclerosis (SSc), systemic lupus erythematosus (SLE), and dermatomyositis (DM). This overlap in clinical presentation is particularly intriguing in medical practice, underscoring the importance of accurately identifying the underlying condition to develop an appropriate treatment plan. Generally, LB is effectively treatable with antibiotics, yet some individuals may develop chronic, non-specific symptoms known as post-treatment Lyme disease syndrome (PTLDS). The similarity of PTLDS symptoms with those of autoimmune, neuromuscular, or other somatic disorders can lead to diagnostic challenges [18].

The related study examined the seropositivity for *B. burgdorferi* in patients with SLE and other rheumatic diseases using ELISA. It analyzed sera from individuals with SLE, seronegative arthritis, RA, and Lyme disease using ELISA and immunoblot tests. The findings revealed seroreactivity to the antigens of *B. burgdorferi* in 40% of SLE patients, 8% of RA patients, and 4% of patients with seronegative arthritis, as determined by ELISA [19]. However, subsequent immunoblot tests did not corroborate these ELISA-positive results. Remarkably, 57% of patients with rheumatoid arthritis showed cross-reactivity to various antigens of Borrelia [19]. This disparity highlights the importance of careful interpretation of positive ELISA results for Lyme disease and the crucial role of immunoblot testing in differentiating true positives from false ones. This study underscores the complexities and limitations of current diagnostic approaches, particularly in populations with autoimmune diseases.

## The possible connection between Borrelia and localized forms of scleroderma and systemic sclerosis

**Fig. 1:**
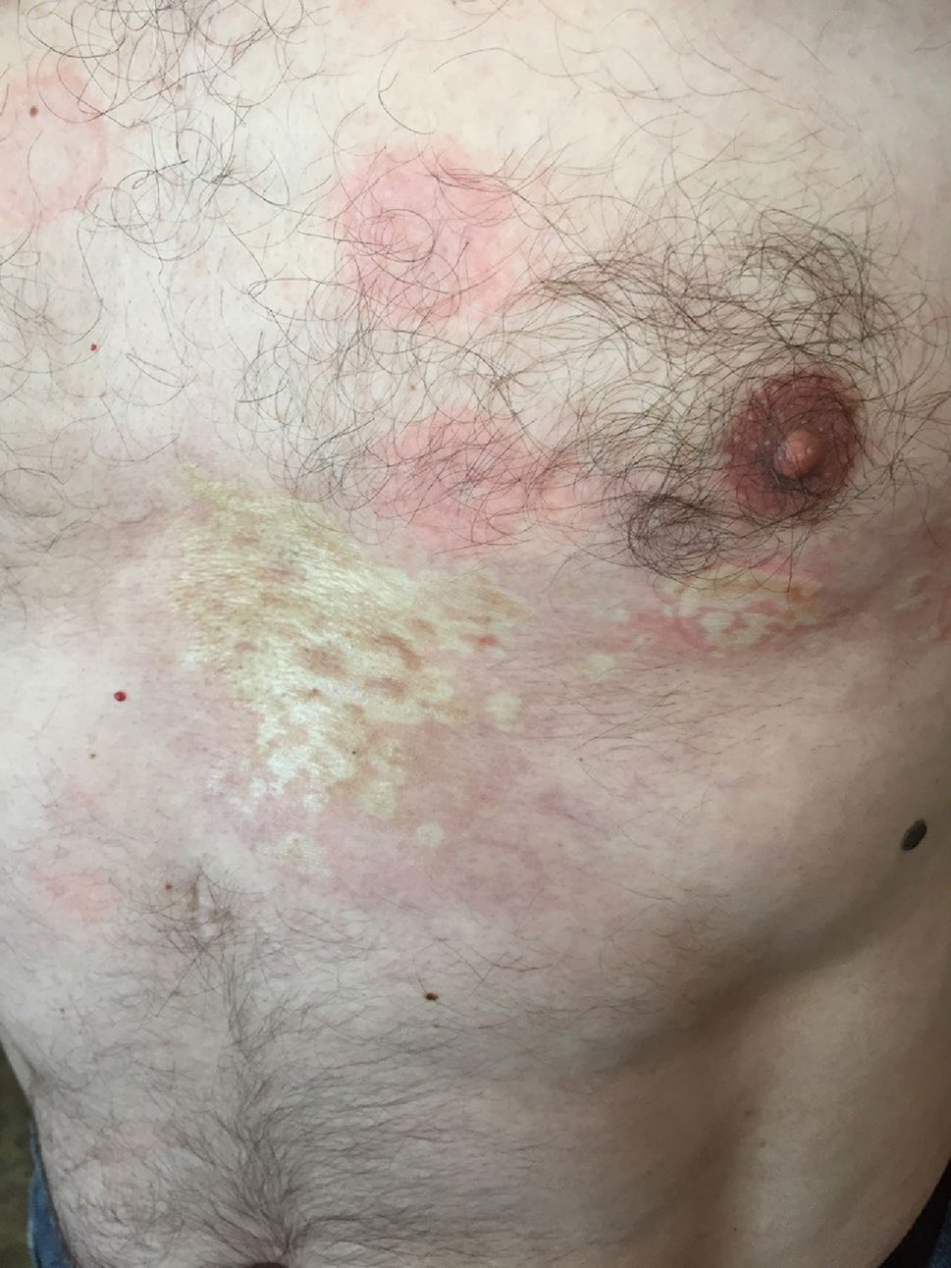
Two isolated, multiform patches of hardened skin on the patient’s chest, who has a medical history of Lyme borreliosis. Patient#1 (The figure is a curtesy of O. Zimba MD)

**Fig. 2:**
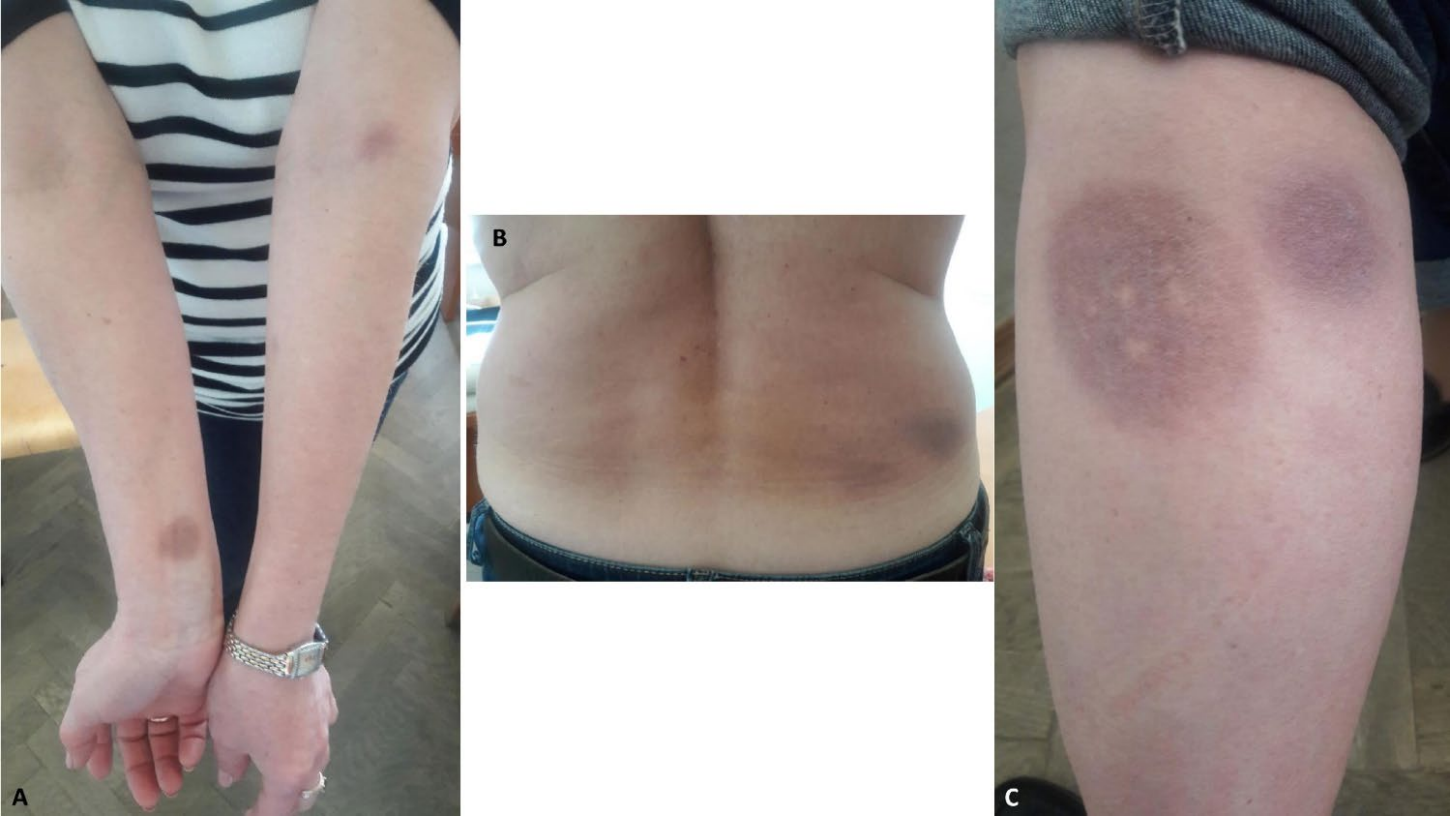
Hyperpigmented and indurated round foci on the skin of the arms (**A**), lower back (**B**), and legs (**C**) which appeared after Lyme borreliosis. Patient#2. (The figure is a curtesy of O. Zimba MD)

Morphea is a localized variant of scleroderma (Figs. 1 and 2). It is identified by the fibrosis of the skin and subcutaneous tissues. [20]. While the exact etiology remains elusive, recent scientific inquiry has delved into the potential association between Borrelia infections and morphea’s development, offering intriguing insights into the complex interplay between infectious agents and autoimmune responses. The hypothesis suggesting a link between Borrelia infection and morphea stems from observations of Borrelia’s ability to trigger autoimmune phenomena [21].

One plausible mechanism involves molecular mimicry, wherein antigens from Borrelia structurally resemble host tissues, leading to cross-reactivity and subsequent autoimmune responses. Borrelia’s surface proteins, particularly those associated with adhesion and evasion of host defenses, may share similarities with skin components, contributing to the development of morphea lesions [3].

Borrelia’s ability to persist within host tissues, evade immune detection, and induce chronic inflammation raises the prospect of sustained immunological responses. In individuals genetically predisposed to autoimmune reactions, the presence of Borrelia may act as a trigger, initiating a series of events that ultimately lead to the fibrotic alterations typical of morphea [22].

While the Borrelia–morphea hypothesis is intriguing, the scientific evidence supporting a direct causal relationship remains inconclusive. Limited studies have explored this association, and conflicting results underscore the need for comprehensive investigations. Some studies report the presence of Borrelia DNA in morphea lesions, while others find no consistent correlation [23].

The emergence of skin lesions in LB is intriguing, particularly when examining the involvement of *B. burgdorferi* in skin lesions that exhibit fibrosis. A striking aspect is the marked affinity of *B. burgdorferi* for collagen and elastic fibers. This feature has been identified in histopathological examination of skin lesions in patients with LB [22].

It is crucial to recognize that *B. burgdorferi* has developed adaptive mechanisms that enhance its ability to invade human tissues, especially those of the musculoskeletal system. Research indicates that decorin, a proteoglycan in ligaments and tendons, is crucial for the bacterium’s invasion process. Experiments have shown that mice lacking decorin exhibit resistance to *Borrelia burgdorferi* infection [24]. Decorin plays a role in the extracellular matrix by binding to collagen types I and II, aiding connective tissue structure. Based on these observations, a scientific theory proposes that when *Borrelia burgdorferi* binds to decorin, it may interfere with its interaction with collagen, potentially causing collagen degradation. This process sheds light on the intricate relationship between *Borrelia burgdorferi* and the connective tissue matrix, and could explain the fibrosis in some skin lesions associated with Lyme borreliosis.

However, challenges in researching the Borrelia–morphea connection include the diversity of Borrelia strains, variations in clinical presentations, and the complexity of autoimmune responses. Future studies should employ advanced molecular techniques, consider host genetics, and address confounding factors to elucidate the mechanisms linking Borrelia infection to morphea.

Given the established causal relationship between *B. burgdorferi* and skin fibrosis that reflects localized scleroderma, the question of a potentially similar relationship with systemic sclerosis arises. The pathogenesis of systemic sclerosis is notably intricate, with a multifaceted etiology that includes altered collagen production by fibroblasts and vascular abnormalities, both contributing to the development of sclerosis [25].

The initial phase in the development of systemic sclerosis is characterized by endothelial injury, often manifested through symptoms like Raynaud's phenomenon and microangiopathy. A combination of genetic, environmental, and infectious elements is thought to influence this condition’s pathogenesis [25]. Chronic infection by *Borrelia burgdorferi* is suspected to affect vascular endothelial cells, causing damage and microvasculopathy and possibly triggering the activation of skin fibroblasts [26, 27]. Moreover, recent study observed a marked increase in collagen mRNA production in fibroblast cultures exposed to Borrelia. Aberer et al. also discovered a significant association between the levels of mRNA for growth factors transforming growth factor beta (TGF-β), platelet-derived growth factor alpha (PDGF-α), calreticulin (CALR), and decorin (DCN) and the synthesis of collagen mRNA [27].

Hosts also utilize various innate and adaptive immune systems to defend against extracellular infectious agents. As part of this defense, infected hosts produce reactive oxygen species (ROS) or reactive nitrogen species (RNS) to combat the infection, a mechanism that has been shown to contribute to the pathogenesis of systemic sclerosis in animal models [28, 29].

A case described by Wackernagel et al. supports the notion of *B. burgdorferi* playing a role in developing systemic sclerosis [30]. They presented a patient exhibiting symptoms such as Raynaud's phenomenon, facial and upper trunk swelling, resulting in skin thickening and sclerosis. Diagnostic tests confirmed the *B. burgdorferi* infection through positive antibody results and PCR in the urine. The patient also tested positive for anti-centromere antibodies. Treatment with intravenous ceftriaxone successfully addressed the infection, leading to total resolution of skin lesions. The discussed paper proposes that *Borrelia burgdorferi* could be considered an etiological factor contributing to systemic sclerosis, particularly in individuals predisposed to its development. However, this hypothesis does not have wide acceptance.

## Borreliosis and systemic lupus erythematosus

Lyme borreliosis and SLE represent distinct medical entities, yet their convergence in clinical presentations underscores the complexity of autoimmune and infectious interactions [31]. The challenge lies in differentiating between LB and SLE, as both can exhibit overlapping symptoms, such as joint pain, fatigue, and neurological complications. The diagnostic process becomes particularly intricate when considering the potential for misinterpretation, especially in regions where LB is endemic. Moreover, the interplay between these conditions raises questions about the potential for *Borrelia burgdorferi* to trigger or exacerbate autoimmune responses, contributing to the development or progression of SLE.

Strisova et al. reported a case involving a 37-year-old woman diagnosed with SLE [32]. The individual experienced multiple misdiagnoses of LB and received treatment with antibiotics of the tetracycline group. Unfortunately, the patient faced a severe worsening of SLE and, ultimately, succumbed to multi-organ failure.

The patient, who had a history of pericarditis, pleuritis, and an acute ischemic stroke, was diagnosed with SLE. However, influenced by online resources, the patient chose to pursue treatment for chronic LB instead of accepting immunosuppressive drugs. This led to repeated serological tests and long-term therapy with Minocycline or Doxycycline over a decade. Despite medical advice, the patient consistently rejected immunosuppressive treatments, resulting in the advancement of systemic autoimmunity and ultimate multi-organ failure.

This case highlights the importance of adhering to established guidelines in diagnosing and treating LB. A positive serology test is not a definitive confirmation of *B. burgdorferi* infection, and long-term antibiotic use for PTLDS can be risky. The Lyme disease self-diagnosed by the patient, coupled with multiple misdiagnoses by medical professionals, led to the refusal of proper immunosuppressive care, culminating in premature death. The case also points out the need for thorough immune system evaluation before beginning IVF procedures, given the potential for thromboembolic events and aggravation of autoimmune diseases.

## Borrelia and dermatomyositis

The link between Lyme disease and dermatomyositis (DM) extends beyond mere coincidence, as evidenced by their co-occurrence in clinical settings. The relationship between these two conditions underscores the intricate nature of LB, where the pathogen’s ability to modulate immune responses and evade detection contributes to a spectrum of manifestations, including autoimmune disorders like DM [33].

Novitch M et al. reported that in contrast to the spontaneous form, DM associated with LB exhibits a higher prevalence in males (five males, three females) [33]. Clinical cases of LD-related DM involve a combination of inherent factors such as age, gender, and comorbidities, as well as external factors like occupation, outdoor activities, residence in areas prone to LB, and delays in treatment. Moreover, a connection has been identified between LB-associated DM and other autoimmune disorders, although the specific contribution of these conditions to the pathogenesis is still unclear. [34–36].

While dermatomyositis (DM) can occur in both acute and chronic LB scenarios, most documented cases are linked to chronic LB infections, possibly due to the prolonged survival of *B. burgdorferi* in the reticuloendothelial system. Numerous efforts were made to comprehend the mechanisms driving the development of DM in LB, indicating a multifaceted pathogenicity [33]. From an immunological perspective, *B. burgdorferi* has the potential to impact both types of immunity. It includes cellular alterations such as lymphopenia, decreased activity of T-suppressor cells, the abnormal ratio between T-helpers and T-suppressors, deviations in natural killer cells and humoral modifications such as an increase in several polyclonal B cells and production of immunoglobulins [3].

Likewise, autoantibodies appear to play a role in cases associated with LB, similar to idiopathic DM. [33]. While there seems to be an inherent inclination to develop DM, not all LB patients experience this complication. DM is generally linked to specific genetic markers, such as HLA-DRB10301/DQA10501 in Caucasians and HLA-B27 in Asians [37]. However, these same genetic associations may not necessarily extend to LB-associated DM. Hoffman et al. propose the potential role of haplotype HLA-Cw3 as a predisposing factor for DM associated with LB, underscoring the intricate interplay between genetics and LB-induced autoimmunity [38].

Misdiagnoses in the context of Lyme borreliosis and autoimmune diseases can inflict significant harm on patients. Inaccurate identification may lead to inappropriate or delayed treatments, allowing the underlying condition to progress unchecked. For instance, mistaking Lyme disease for an autoimmune disorder might result in the unnecessary administration of immunosuppressive therapies, potentially exacerbating the infection. Conversely, misdiagnosing an autoimmune condition as Lyme disease could lead to prolonged antibiotic regimens with associated risks, and the actual autoimmune disorder may remain untreated. Such diagnostic errors may contribute to worsening symptoms, increased morbidity, and, in some cases, fatal outcomes. The toll extends beyond physical health, impacting the patient’s emotional well-being and quality of life [39]. Therefore, precision in diagnostics is crucial to ensure timely and appropriate interventions, minimizing potential harm to those affected by these complex medical conditions.

In conclusion, Borrelia demonstrates a global prevalence and diverse genetic makeup, being the primary cause of Lyme disease. The intricate interplay between Borrelia and the host immunity extends beyond conventional infectious responses, contributing to autoimmune mechanisms such as molecular mimicry and persistent infections. This complexity is reflected in the mimicry of symptoms associated with various autoimmune diseases, including systemic lupus erythematosus, dermatomyositis, local scleroderma, and even systemic sclerosis. Nevertheless, these associations lack a precise explanation and necessitate further investigation. The cases of misdiagnosis between Lyme borreliosis and autoimmune diseases underscore the importance of accurate diagnostics and adherence to guidelines. Understanding Borrelia’s impact on immune responses is crucial for improved diagnostics and targeted therapeutic interventions in Lyme borreliosis and its potential autoimmune implications.

## Data Availability

The data supporting the findings of this study are available within the article.
